# Statin myalgia is not associated with reduced muscle strength, mass or protein turnover in older male volunteers, but is allied with a slowing of time to peak power output, insulin resistance and differential muscle mRNA expression

**DOI:** 10.1113/jphysiol.2014.285577

**Published:** 2015-01-26

**Authors:** Joanne E. Mallinson, Kanagaraj Marimuthu, Andrew Murton, Anna Selby, Kenneth Smith, Dumitru Constantin‐Teodosiu, Michael J. Rennie, Paul L. Greenhaff

**Affiliations:** ^1^ MRC/Arthritis Research UK Centre for Musculoskeletal Ageing ResearchUniversity of NottinghamNottinghamNG7 2UHUK

## Abstract

**Key points:**

- Statins cause muscle‐specific side effects, most commonly muscle aches/weakness (myalgia), particularly in older people. Furthermore, evidence has linked statin use to increased risk of type 2 diabetes. However, the mechanisms involved are unknown.
- This is the first study to measure muscle protein turnover rates and insulin sensitivity in statin myalgic volunteers and age‐matched, non‐statin users under controlled fasting and fed conditions using gold standard methods.
- We demonstrate in older people that chronic statin myalgia is not associated with deficits in muscle strength and lean mass or the dysregulation of muscle protein turnover compared to non‐statin users. Furthermore, there were no between‐group differences in blood or muscle inflammatory markers.
- Statin users did, however, show blunting of muscle power output at the onset of dynamic exercise, increased abdominal adiposity, whole body and leg insulin resistance, and clear differential expression of muscle genes linked to mitochondrial dysfunction and apoptosis, which warrant further investigation.

**Abstract:**

Statins are associated with muscle myalgia and myopathy, which probably reduce habitual physical activity. This is particularly relevant to older people who are less active, sarcopaenic and at increased risk of statin myalgia. We hypothesised that statin myalgia would be allied to impaired strength and work capacity in older people, and determined whether differences aligned with divergences in lean mass, protein turnover, insulin sensitivity and the molecular regulation of these processes. Knee extensor strength and work output during 30 maximal isokinetic contractions were assessed in healthy male volunteers, nine with no statin use (control 70.4 ± 0.7 years) and nine with statin myalgia (71.5 ± 0.9 years). Whole body and leg glucose disposal, muscle myofibrillar protein synthesis (MPS) and leg protein breakdown (LPB) were measured during fasting (≈5 mU^ ^l^−1^ insulin) and fed (≈40 mU^ ^l^−1^ insulin + hyperaminoacidaemia) euglyceamic clamps. Muscle biopsies were taken before and after each clamp. Lean mass, MPS, LPB and strength were not different but work output during the initial three isokinetic contractions was 19% lower (*P *< 0.05) in statin myalgic subjects due to a delay in time to reach peak power output. Statin myalgic subjects had reduced whole body (*P *= 0.05) and leg (*P *< 0.01) glucose disposal, greater abdominal adiposity (*P *< 0.05) and differential expression of 33 muscle mRNAs (5% false discovery rate (FDR)), six of which, linked to mitochondrial dysfunction and apoptosis, increased at 1% FDR. Statin myalgia was associated with impaired muscle function, increased abdominal adiposity, whole body and leg insulin resistance, and evidence of mitochondrial dysfunction and apoptosis.

**Abbreviations**
AA: amino acid
AV: arterialised venous
CHO: carbohydrate
CK: creatine kinase
FDR: false discovery rate
GATM: glycine amidinotransferase
GLM: general linear model
HMBS: hydroxymethylbilane synthase
MPS: muscle protein synthesis
LPB: leg protein breakdown
PCr: phosphocreatine
PDC: pyruvate dehydrogenase complex
PDK: pyruvate dehydrogenase kinase
SAM: significance analysis of microarrays

## Introduction

Statin use has been associated with a 30% reduction in cardiovascular events by reducing cholesterol synthesis (Vaughan & Gotto, 2004). Statins are generally well tolerated, but can have muscle‐specific side effects through a poorly defined mechanism, ranging from myalgia (muscle aches or weaknesses with and without elevated serum creatine kinase (CK) concentration) to, in the severest case, rhabdomyolysis (typically >10 times the upper limit of normal serum CK). Meta‐analysis of randomised controlled trials suggest rhabdomyolysis is rare, although an increased incidence of myalgia is seen in patients receiving statins compared to placebo (McClure *et al*. 2007).

There is currently intense debate as to whether statins should be prescribed to people at low risk of cardiovascular disease (Abramson *et al*. 2013). To date little consideration has been given to whether this could be counter‐productive in the context of physical activity. Specifically, there is overwhelming evidence that physical inactivity, reflected by low aerobic fitness, is a leading cause of morbidity and mortality (Blair *et al*. 2012). Physical inactivity has direct effects on the development of non‐communicable chronic disease, such as cardiovascular disease and type 2 diabetes (WHO, 2010). In clinical practice it has been reported that the incidence of muscle pain in statin users increases with habitual physical activity, with greater numbers of patients avoiding even moderate exertion during every day activities (Bruckert *et al*. 2005). Furthermore, exercise alongside statin therapy has been shown to increase CK levels, muscular cramps and muscular weakness in both sedentary young men (Thompson *et al*. 1997) and professional athletes (Sinzinger & O'Grady, 2003). A recent meta‐epidemiological review suggests that exercise and many drug interventions are similar in terms of their mortality benefits and that exercise interventions should be considered as a viable alternative to, or alongside, drug therapy (Naci & Loannidis, 2013). However, due to the exacerbation of musculoskeletal problems by exercise in statin users this should be viewed with some caution.

The average age of statin users is ∼60 years (Bruckert *et al*. 2005), but it is recommended that people >65 years of age, who are at increased risk of cardiovascular disease, will benefit the most from statin therapy (Grundy *et al*. 2004). For many, however, ageing is accompanied by physical frailty, inactivity, sarcopenia and weakness. Interestingly, older adults taking statins for 2.6 years showed decreased leg strength and increased falls risk compared to age‐matched controls (Scott *et al*. 2009). Furthermore, animal studies suggest statin myopathy is associated with decreased muscle force (Fuzi *et al*. 2012), reduced aerobic capacity and impaired mitochondrial respiration in skeletal muscle (Bouitbir *et al*. 2011). Conversely, Parker *et al*. (2012) demonstrated that 6 months of atorvastatin treatment (80 mg day^−1^) in healthy individuals across three age ranges (20–39, 40–54 and ≥55 years) had no greater impact on muscle strength than placebo, although when comparing these asymptomatic subjects on atorvastatin with myopathic volunteers, isometric leg strength was significantly lower in the latter. Given this lack of literature concordance, we aimed to determine whether people >65 years of age prescribed statins for ∼4 years, and experiencing muscle‐related aches and pains, presented with impaired muscle function (both strength and fatigability) and exacerbated muscle mass loss, and also combine this with comprehensive metabolic and molecular phenotyping of these same individuals.

There are a number of theories regarding the mechanistic basis of statin‐mediated muscle dysfunction. In short, evidence suggests statin‐mediated dysregulation of PI3k/Akt and downstream signalling targets may play a major role in perturbation of muscle protein and carbohydrate (CHO) metabolism (Mallinson *et al*. 2009), and may explain the muscle weakness and fatigability reported in statin myopathic individuals. Furthermore, pharmacologically increasing muscle CHO oxidation by administering the pyruvate dehydrogenase kinase (PDK) inhibitor dichloroacetate concurrently with simvastatin abolished statin myopathy *in vivo* in rodents. This occurred, at least in part, via inhibition of FOXO‐mediated transcription of genes regulating muscle CHO utilisation and protein breakdown (Mallinson *et al*. 2012). Additionally, meta‐data analyses associated intensive statin therapy (up to 40 mg day^−1^) with increased risk of new‐onset diabetes compared to moderate dose therapy (Preiss *et al*. 2011) and a single nucleotide polymorphism of 3‐hydroxy‐3‐methylglutaryl–CoA reductase (HMG–CoA, the target of statin therapy) has also been linked to increased risk of type 2 diabetes (Swerdlow *et al*. 2014). Indeed, regular statin therapy reduces insulin sensitivity and glucose disposal (Larsen *et al*. 2013). It is possible therefore that dampened PI3k/Akt signalling may be directly related to the statin‐mediated decrease in insulin sensitivity reported in humans. Alternatively, given that physical inactivity induces insulin resistance, exemplified by data demonstrating a 2 week transition from an ambulatory lifestyle to an inactive state induces insulin resistance and increases central adiposity in healthy volunteers (Olsen *et al*. 2008), it is not unreasonable to conclude that impaired insulin sensitivity associated with statin use could result directly from a statin‐mediated decrease in physical activity levels. Indeed, as outlined above, Bruckert *et al*. (2005) reported that patients experiencing statin‐related muscle pain avoided even moderate exertion during every day activities

In the present study we therefore determined whether older people chronically prescribed statins and reporting myalgia symptoms presented with (1) reduced muscle mass, strength and work output during repeated maximal contractions and (2) elevated leg protein breakdown in the fasted state and, when exposed to a ‘fed state’ (hyperaminoacideamia, hyperinsulinaemia) clamp, and showed (3) a blunted increase in muscle protein synthesis and dampened decrease in muscle protein breakdown and (4) insulin resistance compared to age‐matched control volunteers. We also hypothesised dysregulation of these physiological responses would be accompanied by changes in associated molecular pathways in muscle.

## Methods

### Ethical approval

This study was approved by the University of Nottingham Medical School Ethics Committee in accordance with the Declaration of Helsinki. Before taking part, all subjects gave informed consent.

### Subjects

Nine healthy men (70.4 ± 0.7 years) with a history of continuous statin use of 4 ± 0.5 years (statin myalgic subjects) and nine age‐ (71.5 ± 0.9 years) and sex‐matched volunteers with no history of statin use (control) participated in this study. Before taking part, all subjects underwent routine medical screening and completed a general health questionnaire. Volunteers reporting any form of chronic muscle pain since starting statin therapy were recruited into the study. The presence of statin myalgia was confirmed by measuring plasma CK concentration and by physical examination and completion of a muscle pain questionnaire. The questionnaire was based on the short form McGill pain questionnaire (Melzack, 1975) and included questions relating to the characteristics of muscle pain (location, frequency, type and intensity, triggering and aggravating factors, and impact of pain on daily activities). Twenty‐four potential myalgic volunteers were screened and nine were deemed to be suitable for inclusion into the study. Inclusion criteria included no evidence of chronic endocrine, renal or respiratory disease; diastolic blood pressure no higher than 95 mmHg, normal blood clotting and smoking‐free over the past 3 years. Statin myalgic subjects were included if plasma CK increased above normal values or they reported muscle pain not related to an incident and since commencing statin therapy (determined from the questionnaire). Subjects on medication apart from statins that affect muscle metabolism were excluded from the study. Subject characteristics are presented in Table 1 and detailed information on statin myalgic subjects in Table 2.

**Table 1:** **Physical and clinical characteristics of control and statin user groups**

Characteristic	Control (*n* = 9)	Statin (*n* = 9)	*P*
Age (years)	71.5 ± 0.9	70.4 ± 0.7	NSD
Height (cm)	175.1 ± 3.0	174.1± 2.9	NSD
Weight (kg)	76.7 ± 3.7	81.2 ± 4.1	NSD
Statin use (years)	0	4 ± 0.5	—
Statin type:			
Simvastatin	0	*n* = 8	
Lipitor	0	*n* = 1	
Muscle soreness score	1	4 ± 1.25	<0.001
Plasma CK (U l^–1^)	114 ± 12.8	92 ± 11.3	NSD
Plasma LDH (U l^–1^)	359 ± 23.6	348 ± 14.5	NSD
Muscle total creatine (mmol kg dry muscle^−1^)	117.2 ± 5.8	108.4 ± 6.2	NSD
Muscle ATP (mmol kg dry muscle^−1^)	18.7 ± 1.1	18.5 ± 1.1	NSD
Plasma TNFα (pg ml^−1^)	1.0 ± 0.2	1.2 ± 0.2	NSD
Plasma IL6 (pg ml^−1^)	2.3 ± 0.5	2.8 ± 0.4	NSD

Values are expressed as mean ± SEM and score data are expressed as median ± interquartile range. Muscle soreness score is based on a scale of 1–10 with 1 being no soreness and 10 being worse possible soreness. CK, creatine kinase; LDH, lactate dehydrogenase; TNFα, tumour necrosis factor alpha; IL6, interleukin 6; NSD, no significant difference.

**Table 2:** **Statin myalgia subject characteristics**

Subject	Age (years)	Statin/dose (mg)	Duration on statin (years)	Duration of myalgia	Muscle soreness score*	Exacerbate symptoms	Alleviate symptoms	Concurrent medications
1	68	Simvastatin/40	5	>1 year	4	Walking	Rest	Ramipril, aspirin
2	73	Simvastatin/20	6	>1 year	2	No response	No response	Omeprazole
3	73	Simvastatin/20	2	>1 year	4	Walking	Rest, pain	Ramipril, aspirin
							killers	
4	68	not provided	10	2 months	3	No response	No response	Atenolol
5	71	Simvastatin/40	1	>1 year	4	Walking	Rest	None
6	71	Simvastatin/40	4	>1 year	7	No response	No response	None
7	68	Simvastatin/40	12	>1 year	5	Standing, lifting	Rest	Perindopril, aspirin, nicotinic acid
8	70	Atorvastatin/80	3	>1 year	2	Lifting, walking	Rest	Ramipril, aspirin
9	70	Simvastatin/40	5	>1 year	3	Activity	Rest, pain	Omeprazole,
							killers	bendroflumethiazide,
								ramipril

*Muscle soreness was assessed on a scale of 0 to 10, with 0 being no soreness and 10 being the worse possible soreness.

### Body composition, muscle isometric strength and dynamic muscle function

Volunteers underwent a dual‐energy X‐ray absorp‐tiometry scan to determine whole body and regional lean and fat masses (Lunar Prodigy; GE Healthcare, Little Chalfont, UK). Isometric strength was determined (best of three attempts) in the knee extensor muscles during a static maximal voluntary contraction using an isometric knee extension unit (Medical Physics, University of Nottingham, UK). In addition, peak power output and work output during 30 maximal voluntary isokinetic knee extensions (at a speed of 90 deg s^–1^) were determined using an isokinetic dynamometer (Isocom, Eurokinetics, Nottingham, UK). Subjects were seated securely in the dynamometer chair with the knee flexed at 90 deg, the ankle fixed to the lever arm and the actuator axis aligned to the lateral femoral epicondyle. Subjects were instructed to push as hard as possible against the lever arm until full extension was reached and to relax upon flexion until 30 contractions had been completed.

### Experimental protocol

The protocol (Fig.1) was designed to allow the measurement of muscle protein synthesis (by incorporation of [1,2‐^13^C]leucine into quadriceps muscle protein) and leg protein breakdown (as the rate of dilution of [^2^H_5_]phenylalanine) in the fasted (target serum insulin concentration of 5 mU l^−1^) and fed state (target serum insulin concentration of 40 mU l^−1^ and a constant infusion of mixed amino acids (AAs) at 10 g h^−1^) (Rennie *et al*. 1982). All subjects were asked to abstain from alcohol and exercise for 48 h prior to the experimental visit and to arrive in the morning in a fasted state. Subjects rested semi‐supine before a femoral vein cannula (ES‐04150; Arrow Deutschland, Neu‐Isenburg, Germany) was inserted for leg venous blood sampling. A venous cannula was retrogradely inserted into a superficial vein on the dorsal surface of the non‐dominant hand which was kept in a hand‐warming unit (air temperature: 50–55°C) to arterialise the venous drainage of the hand (Gallen & Macdonald, 1990). Cannulas were also sited at the antecubital fossa in both forearms for infusion of mixed AAs as well as octreotide, glucagon, glucose and insulin to clamp insulin concentrations at various values. Patency of the lines was maintained using 0.9% NaCl infusions (Baxter Healthcare, Newbury, UK).

**Figure 1:**
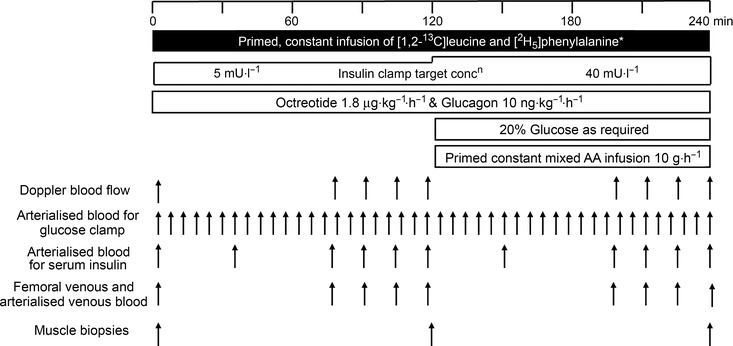
**Study protocol** Primed constant infusions of stable isotope‐labelled AAs ([^2^H_5_]phenylalanine and [1,2‐^13^C] leucine) were administered for a total period of 240 min. Serum insulin was maintained at fasting concentrations (≈5 mU l^−1^) for 120 min and endogenous insulin production was suppressed by infusion of octreotide, and post‐absorptive glucagon concentration was maintained by infusion of glucagon. After 120 min, serum insulin was raised equivalent to a fed state (≈40 mU l^−1^). Quadriceps muscle biopsies were taken at baseline, 120 min and 240 min.

Primed constant infusions of stable isotope labelled AAs [^2^H_5_]phenylalanine and [1,2‐^13^C_2_]leucine (Cambridge Isotope Laboratories, Tewksbury, MA, USA) were administered for a total period of 240 min. At the start of the tracer administration, serum insulin was maintained at fasting concentrations (≈5 mU l^−1^) by the administration of insulin (0.6 mU m^−2^ min^−1^; human Actrapid, Novo, Denmark), endogenous insulin production was suppressed by infusion of octreotide (1.8 μg kg^−1^ h^−1^; Sandostatin, Novartis, Switzerland) and post‐absorptive glucagon concentration was maintained by infusion of glucagon (10 ng kg^−1^ h^−1^). After 120 min, serum insulin was raised equivalent to the fed state (≈40 mU l^−1^) by the infusion of 40 mU m^−2^ min^−1^ insulin. Blood glucose concentration was measured at 5 min intervals in arterialised venous (AV) blood samples, and a variable infusion of 20% glucose (Baxter Healthcare, Newbury, UK) was initiated to maintain a plasma glucose concentration of 4.5 mmol l^−1^ during the insulin infusion. Concurrent with the period of elevated serum insulin, a primed, constant infusion of mixed AA (Glamin, Fresenius‐Kabi, Runcorn, UK) was administered at 70 ml h^−1^ to deliver 10 g h^−1^ of AAs. AV and femoral venous blood was collected at 0, +30, +90, +105, +120, +150, +195, +210, +225 and +240 min for the analysis of blood glucose and serum insulin (AV only). Femoral arterial blood flow, and femoral venous and AV blood samples were taken at 0, +75, +90, +105, +120, +195, +210, +225 and +240 min for blood tracer measurements. Vastus lateralis muscle biopsies were obtained from the leg in which the femoral vein cannula was placed using the percutaneous needle biopsy technique (Bergstrom, 1975) at 0, 120 and 240 min, and were snap frozen and stored in liquid nitrogen. Following insulin infusion for 240 min, glucose infusion was continued for ∼1 h to stabilise blood glucose concentration, and after which the subjects were provided with a meal.

### Blood glucose and serum insulin

Glucose concentration was measured on AV and femoral venous blood samples using a glucose analyser (YSI 2300 STATplus; Yellow Springs Instruments, Yellow Springs, OH, USA) and serum insulin was measured on AV blood with an ELISA kit (DX‐EIA‐2935, DRG Instruments GmbH, Marburg, Germany).

### Muscle metabolites

One portion of each biopsy sample was freeze dried and dissected free of visible blood and connective tissue, powdered and used for the determination of muscle ATP, phosphocreatine (PCr) and free creatine, using a modification of the spectrophotometric method of Harris (1974).

### Muscle pyruvate dehydrogenase complex (PDC) activity

A small portion of frozen ‘wet’ muscle was used to determine PDC activity as previously described (Constantin‐Teodosiu *et al*. 1991). Briefly, the activity of PDC in its dephosphorylated active form (PDCa) was assayed in a buffer containing NaF and dichloroacetate, and was expressed as a rate of acetyl‐CoA formation (mmol min^−1^ kg wet muscle^−1^) at 37°C.

### Muscle mRNA expression

RNA was extracted from ∼30 mg snap‐frozen muscle as previously described (Mallinson *et al*. 2009). Taqman low density arrays were performed in the ABI PRISM 7900HT sequence detection system and SDS 2.1 software (Applied Biosystems, Foster City, CA, USA). Ninety‐six genes from pathways involved in CHO and fat metabolism, myogenesis, insulin signalling, proteolysis, autophagy, apoptosis, inflammation and associated transcription factors were determined. The candidate genes were chosen from PubMed literature searches and data obtained from our laboratory (Stephens *et al*. 2013). A complete list of details for each gene is provided in Table 3. Data were analysed using RQ Manager software (Applied Biosystems) where the threshold level was normalised across all plates before *C*_t_ values were calculated for each gene and sample. Relative quantification of the genes of interest was measured using the ΔΔ*^C^* ^t^ method with hydroxymethylbilane synthase (HMBS) as the endogenous control. *C*_t_ values for HMBS were not different between control and statin groups (data not shown). Differentially expressed genes were identified by significance analysis of microarrays (SAM) using false discovery rates (FDRs) of 5 and 1% and the maximum number of permutations (24310) (MeV 4.5, TM4).

**Table 3:** **List genes used on low density RT‐PCR array microfludic cards (Applied Biosystems Inc., Foster City, CA, USA)**

**Pathway**	**Gene**	**Protein**	**ABI code**
Carbohydrate metabolism	CKM	Creatine kinase	Hs00176490_m1
	LDHB	Lactate dehydrogenase B	Hs00929956_m1
	NDUFS4	NADH dehydrogenase (ubiquinone) Fe–S protein4	Hs00942568_m1
	PDK4	Pyruvate dehydrogenase kinase, isoenzyme 4	Hs00176875_m1
	SLC2A4	GLUT4	Hs00268200_m1
Fat metabolism	SLC22A5	Carnitine transporter OCTN2	Hs00161895_m1
	CPT1B	Carnitine palmitoyl transferase 1	Hs00993896_g1
	FABP3	Fatty acid binding protein 3	Hs00269758_m1
	FASN	Fatty acid synthase	Hs00188012_m1
	HSPA5	Heat shock protein 70 kDa 5	Hs99999174_m1
	NDUFA5	NADH dehydrogenase [ubiquinone] 1 alpha subcomplex subunit 5	Hs00606273_g1
	PRKCA	Protein kinase C alpha	Hs00925195_m1
	PRKCE	Protein kinase C epsilon	Hs00178455_m1
	PRKCI	Protein kinase C iota	Hs00702254_s1
	PRKCQ	Protein kinase C theta	Hs00989970_m1
	SCD	Stearoyl‐coenzyme A desaturase 1	Hs01682761_m1
	FAT	Fatty acid translocase/CD36	Hs00169627_m1
	FDPS	Farnesyl diphosphate synthase	Hs00266635_m1
Insulin signalling	Akt2	Protein kinase B/Akt, isoform 2	Hs01086102_m1
	EIF2B2	Eukaryotic translation initiation factor 2B, subunit 2 beta	Hs00204540_m1
	MAP2K1	Mitogen‐activated protein kinase kinase 1	Hs00605615_mH
	MAPK3	Mitogen‐activated protein kinase 3	Hs00385075_m1
	PIK3R1	Phosphatidylinositol 3‐kinase, regulatory 1 (p85 alpha)	Hs00933163_m1
	PRKAA1	A‐Kinase, cAMP‐dependent protein kinase,	Hs01562308_m1
	RASA1	p21 Ras	Hs00243115_m1
	RHOQ	Ras homologue gene family, member Q	Hs00817629_g1
	RRAGA	Ras‐related GTP binding A	Hs00538975_s1
Oxidative phosphorylation/ETC	COX7A1	Cytochrome c oxidase subunit VIIa polypeptide 2	Hs00156989_m1
	CYCS	Cytochrome c	Hs01588974_g1
	MT‐CO1	COX‐I, Cytochrome c oxidase I	Hs02596864_g1
	MT‐CO2	COXII, Cytochrome c oxidase 2	Hs00153133_m1
	UQCRQ	Coenzyme Q‐cytochrome c reductase, complex III	Hs00416927_g1
	SDHA	Complex II, succinate dehydrogenase	Hs00188166_m1
	CoQ10B	Ubiquinone/CoQ10	Hs00257861_m1
Transcription factor	FOXO1A	Forkhead transcription factor 1A	Hs00231106_m1
	FOXO3	Forkhead transcription factor 3	Hs00818121_m1
	MEF2C	Myocyte specific enhancer factor 2	Hs00231149_m1
	MYOD1	Myogenic factor 3	Hs00159528_m1
	MYOG	Myogenin	Hs00231167_m1
	PPARA	Peroxisome proliferator‐activated receptor alpha	Hs00231882_m1
	PPARD	Peroxisome proliferator‐activated receptor beta	Hs00602622_m1
	PPARG	Peroxisome proliferator‐activated receptor gamma	Hs01115513_m1
	PPARGC1A	Peroxisome proliferator‐activated receptor‐gamma coactivator 1 alpha	Hs01016724_m1
	RELA	NF‐kappaB3	Hs00153294_m1
	SREBF2	Sterol regulatory element binding protein 2	Hs01081778_m1
	SREBP1c	Sterol regulatory element binding protein 1c	Hs01088691_m1
	ATF3	Activating transcription factor 3	Hs00231069_m1
Transcription factor	NFKB1	Nuclear factor of kappa light polypeptide gene enhancer in B‐cells 1	Hs00765730_m1
	NFKB2	Nuclear factor of kappa light polypeptide gene enhancer in B‐cells 2	Hs01028894_m1
	IKKbeta	Inhibitor of kappa light polypeptide gene enhancer in B‐cells, kinase beta	Hs00233287_m1
	AP1	jun proto‐oncogene	Hs00277190_s1
Myogenesis	ROCK1	ROCK	Hs00178463_m1
	RHOA	Ras homolog gene family, member A	Hs00236938_m1
	RHOB	Ras homolog gene family, member B	Hs00269660_s1
	MYF5	Myogenic factor 5	Hs00271574_m1
	HRAS	Transforming protein p21	Hs00610483_m1
	MET	Met proto‐oncogene	Hs01565584_m1
	CDH15	m‐cadherin 15	Hs00170504_m1
	RHEB	Ras homolog enriched in brain	Hs00950800_m1
	CD34	CD34	Hs00990732_m1
Autophagy	GABARAP	GABA(A) receptor‐associated protein	Hs00925899_g1
	TFAM	Transcription factor A, mitochondrial	Hs01082775_m1
	BNIP3	BCL2/adenovirus E1B 19 kDa interacting protein 3	Hs00969291_m1
	DRP1	Dynamin 1‐like	Hs00247147_m1
Proteolysis	CAST	Calpastatin	Hs00156280_m1
	UBB	Ubiquitin‐B	Hs00430290_m1
	UBC	Ubiquitin‐C	Hs00824723_m1
	MAFbx	F‐box protein 32	Hs01041408_m1
	TRIM63	MuRF1	Hs00261590_m1
	PSMA beta 1	Proteasome subunit beta type 1	Hs00427357_m1
	PSMA beta 2	Proteasome subunit beta type 2	Hs01002946_m1
	PSMA beta 5	Proteasome subunit beta type 5	Hs00605652_m1
	USP19	Ubiquitin specific peptidase 19	Hs00324123_m1
	CAPN1	Calpain 1	Hs00559804_m1
	CAPN2	Calpain 2	Hs00965092_m1
	CAPN3	Calpain 3	Hs00544975_m1
	MSTN	Myostatin	Hs00976237_m1
	CTSL1	Cathepsin L	Hs00377632_m1
	MMP2	Matrix metallopeptidase 2	Hs01548727_m1
Apoptosis	CASP9	Caspase‐9	Hs00154261_m1
	CASP8	Caspase‐8	Hs01018151_m1
	Smac/Diablo	Diablo IAP‐binding mitochondrial protein	Hs00219876_m1
	APAF‐1	Apoptotic peptidase activating factor 1	Hs00559441_m1
	BCL2	B‐cell CLL	Hs00608023_m1
Inflammation	TNF	Tumour necrosis factor α	Hs99999043_m1
	Il‐6	Interleukin‐6	Hs00985639_m1
	MT1A	Metallothionein 1A	Hs00831826_s1
Calcium release	RYR1	Ryanodine receptor 1	Hs00166991_m1
	RYR3	Ryanodine receptor 3	Hs00168821_m1
Organic anion transporter	SLCO1B1	Solute carrier organic anion transporter family, member 1B1	Hs00272374_m1
Housekeeping gene	ACTA1	Actin, alpha	Hs00559403_m1
	HMBS	Hydroxymethylbilane synthase	Hs00609297_m1
	B2M	Beta2‐microglobulin	Hs00187842_m1
	POLR2A	RNA polymerase 2a	Hs01108291_m1

### Western blotting

Protein was extracted from ∼30 mg snap‐frozen tissue as previously described (Mallinson *et al*. 2012). Total and phosphorylated 4EBP1 (Ser^65^) and p70S6k (Thr^389^) (Cell Signaling, Danvers, MA, USA) and PDK4 (Millipore, UK) were analysed with anti‐mouse IgG (H+L) (DyLight 800 Conjugate, Cell Signaling) or anti‐rabbit IgG (H+L) (DyLight 680 Conjugate, Cell Signaling) as the secondary antibodies. Blots were scanned and bands identified using the Odyssey Infrared Imaging System (LI‐COR Biosciences, Lincoln, NE, USA). Dual probing was used for total and phosphorylated proteins. Density volume was adjusted by subtracting the local background and then normalised with β‐actin (Sigma Aldrich, Dorset, UK).

### Leucine incorporation into myofibrillar muscle protein

Myofibrillar leucine incorporation into muscle was deter‐mined using capillary gas chromatography–combustion‐–isotope‐ratio mass spectrometry (GC‐C‐IRMS) using our standard techniques (Kellis & Baltzopoulos, 1998).

### Measurement of tissue free and plasma and AA enrichment and concentration

Intracellular leucine enrichment was determined by GC‐MS as described previously (Burd *et al*. 2010). To determine labelling (atom % excess) of plasma leucine and phenylalanine, plasma proteins were precipitated with ice cold ethanol, and the supernatant, containing free amino acids dried under N_2_, was solubilised in 0.1 m HCl and the lipid fraction extracted into ethyl acetate. The aqueous layer was then dried under nitrogen and the *tert*‐butyldimethylsilyl (*t‐*BDMS) derivative formed for analysis by GC‐MS (MD800; Fisons, Ipswich, UK) using electron impact ionisation with selected ion monitoring. Blood leucine and phenylalanine concentrations were determined using GC‐MS with reference to the appropriate standard curves.

### Calculations

#### Whole body glucose disposal

Whole body glucose disposal (mg kg body mass^−1^ min^−1^) was calculated during the fed state clamp once a steady state had been reached (195–240 min) from the rate of glucose infusion averaged over 5 min periods as described by DeFronzo *et al*. (1979).

#### Leg glucose uptake

Leg glucose uptake (mg kg leg lean mass^−1^ min^−1^) was calculated during the fed state clamp once a steady state had been reached using the equation (AV glucose concentration – venous glucose concentration) × leg blood flow/leg lean mass.

#### Muscle protein synthesis (MPS) or fractional synthetic rate

The rate of MPS between the biopsies was calculated using the standard equation of fractional protein synthesis (% h^−1^), ∆*E*_m_/*E*_p_ (1/*t*)  × 100, where ∆*E*_m_ is the change in labelling of muscle protein leucine between two biopsy samples, *E*_p_ is the mean enrichment over time of the precursor for protein synthesis (taken as intraceullar leucine) and *t* is the time between biopsies.

#### Leg protein breakdown (LPB)

LPB was calculated from the AV dilution of [^2^H_5_]phenylalanine tracer AA using the following equation: [(*E*_a_/*E*_v_) – 1] × 2*C*_v_ × BF, where *E*_a_ and *E*_v_ are the values of phenylalanine labelling at steady state in AV and femoral venous blood, respectively, *C*_v_ is the concentration of phenylalanine in femoral venous blood, and BF is the blood flow in ml per leg. Leg lean mass (kg) was calculated from the dual‐energy X‐ray absorptiometry scans.

### Statistics

All data are expressed as mean ± SEM. An independent samples Student's *t*‐test was used to compare differences between groups. For repeated measures, a repeated measures general linear model (GLM) was used and a least significance difference *post hoc* test was performed to locate significant differences. Score data were analysed with a Mann Whitney *U* test and a Pearson's correlation coefficient was used to determine any relationship between fat mass and glucose disposal rate. A significance level of *P *< 0.05 was used. All calculations were performed using IBM SPSS 19.0.

## Results

### Physical characteristics, clinical chemistry and body composition

Clinical chemistry showed no significant differences in plasma CK, lactate dehydrogenase (LDH), interleukin 6 (IL6) or tumour necrosis factor α (TNFα) levels between groups (Table 1), although the magnitude of perceived muscle soreness was 4‐fold greater in the statin myalgic subjects (*P *< 0.001). Muscle total creatine and ATP content was also similar between control and statin myalgic subjects (Table 1).

There was no difference in regional (arm, trunk, leg) lean tissue mass (Fig. 2) when comparing control and statin myalgic subjects. However, arm and trunk fat mass was significantly greater in statin myalgic subjects (Fig. 2, *P *< 0.05).

**Figure 2:**
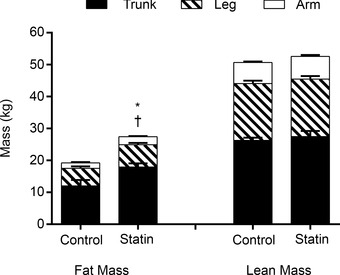
**Body composition of control and statin user groups** Values are expressed as mean ± SEM. *P < 0.05 when comparing arm fat mass, and †*P *< 0.05 when comparing trunk fat mass between control and statin myalgic subjects.

### Muscle functional measurements

Isometric strength of the knee extensors was not different between control and statin myalgic subjects (Fig. 3 *A*). Similarly, peak power output during 30 maximal knee extensor contractions was not different between groups (Fig. 3 *B*). However, whilst the control group achieved peak power at the third contraction, the statin user group achieved peak power output at the twelfth contraction, producing significantly less work output per contraction at the onset of exercise (Fig. 3 *C*, *P *< 0.05).

**Figure 3:**
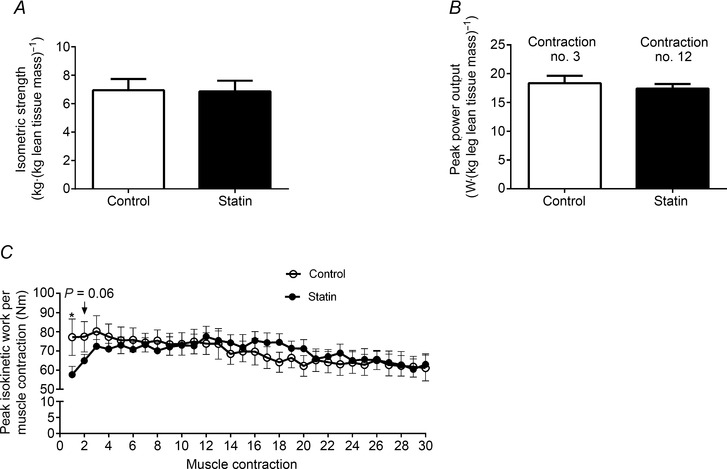
**Muscle functional measurements in control and statin user groups** *A*, isometric strength (kg kg leg lean mass^−1^); *B*, peak power output (W kg leg lean mass^−1^); *C*, peak isokinetic work per contraction (Nm) during 30 maximal isokinetic contractions. Values are expressed as mean ± SEM. *P < 0.05 compared to control.

### Serum insulin concentration, whole body and leg glucose disposal, and muscle pyruvate dehydrogenase complex regulation

When serum insulin was clamped at a fasting concentration for 2 h (insulin infusion rate of 0.6 mU m^2^ min^−1^), no difference in steady‐state serum insulin concentration was seen when comparing control and statin user groups (data not shown). Similarly, when the insulin infusion rate was increased to 40 mU m^2^ min^−1^, along with AA infusion at 10 g h^−1^ to create a fed state condition, no difference in steady‐state serum insulin concentration was observed, although there was a trend for the area under the steady‐state insulin curve to be greater in the statin user group (Fig. 4 *A*, *P *= 0.07), probably reflecting blunted insulin clearance in statin myalgic subjects, given that endogenous insulin production was suppressed by octreotide infusion (see Methods). The rate of whole body glucose disposal during the fed state insulin clamp was 30% lower in the statin user group compared to control (Fig. 4 *B*, *P *= 0.05) and was accompanied by leg glucose uptake being 45% lower in the statin group (Fig. 4 *C*, *P *< 0.01). A negative correlation was seen between whole body glucose disposal and trunk fat mass (Fig. 4 *D*, *r* = –0.70, *P *< 0.01). The fed state insulin clamp tended to increase muscle PDC activity, but this was not significantly different from baseline or between groups (Table 4). Muscle PDK4 protein expression was not different between control and statin myalgic subjects at baseline or after both fasted and fed state insulin clamps (Table 4).

**Figure 4:**
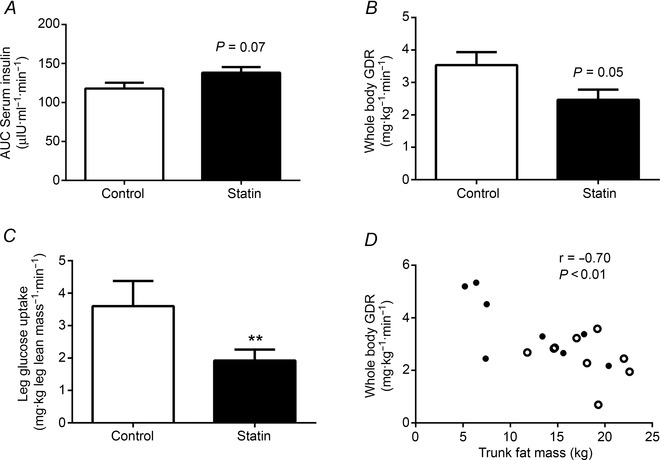
**Serum insulin concentration and blood glucose disposal rate in control and statin user groups** *A*, serum insulin area under the curve (AUC) (μIU ml^−1^ min^−1^) during the period of steady‐state glucose disposal of the fed state clamp (40 mU m^−2^ min^−1^ insulin and 10 g h^−1^ mixed amino acids); *B*, rate of steady‐state whole body glucose disposal (mg kg^−1^ min^−1^) during the fed state clamp; *C*, rate of steady‐state leg glucose uptake during the fed state clamp (mg kg^−1^ leg lean mass min^−1^); *D*, relationship between whole body glucose disposal (mg kg^−1^ min^−1^) and trunk fat mass (kg). Closed circles = control, open circles = statin myalgic subjects. All values are expressed as mean ± SEM. **P < 0.01 compared to control.

**Table 4:** **Muscle PDC activity and PDK4 protein expression in control and statin user groups at baseline, after a 2 h fasted state insulin clamp and a 2 h fed state insulin clamp**

	Control (*n* = 9)	Statin (*n* = 9)
PDC activity (mmol^ ^min^−1 ^kg^−1^)		
Baseline	0.55 ± 0.08	0.41 ± 0.09
Fasted clamp	0.49 ± 0.09	0.41 ± 0.07
Fed clamp	0.67 ± 0.12	0.57 ± 0.13
PDK4 protein expression (mean intensity)		
Baseline	0.61 ± 0.17	0.37 ± 0.05
Fasted clamp	0.45 ± 0.09	0.44 ± 0.05
Fed clamp	0.59 ± 0.24	0.51 ± 0.07

All values expressed as mean ± SEM. Within‐ and between‐group effects are all non‐significant.

### MPS and LPB

The rate of MPS was similar between control and statin myalgic subjects during the fasting insulin clamp (Fig. 5 *A*). As expected, the fed state clamp nearly doubled MPS (*P *< 0.001 for both groups), but the magnitude of the increase was not different between groups (Fig. 5 *A*). LPB was not different between the control and statin myalgic subjects during the fasting insulin clamp (Fig. 5 *B*). Furthermore, no statistical difference was seen when comparing treatment groups during the fed state insulin clamp, although the average reduction in LPB by insulin was greater in the statin myalgic subjects (Fig. 5 *B*), probably reflecting the trend for steady‐state serum insulin concentration to be greater in this group (Fig. 4 *A*).

**Figure 5:**
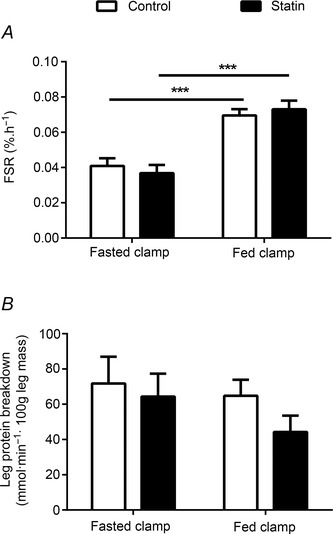
**Muscle protein synthesis and leg protein breakdown in control and statin user groups** *A*, rate of muscle protein synthesis (fractional synthetic rate, FSR) during a 2 h fasted state insulin clamp (0.6 mU m^−2^ min^−1^ insulin) and a 2 h fed state insulin clamp (40 mU m^−2^ min^−1^ insulin and 10 g h^−1^ mixed AAs); *B*, rate of leg protein breakdown during a 2 h fasted state and a 2 h fed state insulin clamp. Values are expressed as mean ± SEM. ***P < 0.001 when fasted *vs*. fed clamp.

### Muscle anabolic signalling

There was no change in the ratio of muscle phosphorylated to total p70S6k protein expression from basal during the fasted state insulin clamp when comparing control and statin myalgic subjects. The fed state clamp tended to increase p70S6k protein phosphorylation in statin myalgic subjects above control (*P *= 0.08, Fig. 6 *A*). Similarly, there was no change from basal in the ratio of phosphorylated to total 4EBP1 protein expression during the fasted state insulin clamp in both experimental groups. However, 4EBP1 phosphorylation was significantly greater in the statin myalgic subjects at the end of the fed state clamp relative to the control group (*P *< 0.05, Fig. 6 *B*). The greater phosphorylation status of p70S6k and 4EBP1 at the end of the fed state clamp in the statin myalgic subjects relative to control probably reflects the trend for steady‐state serum insulin concentration to be greater in the statin group (Fig. 4 *A*).

**Figure 6:**
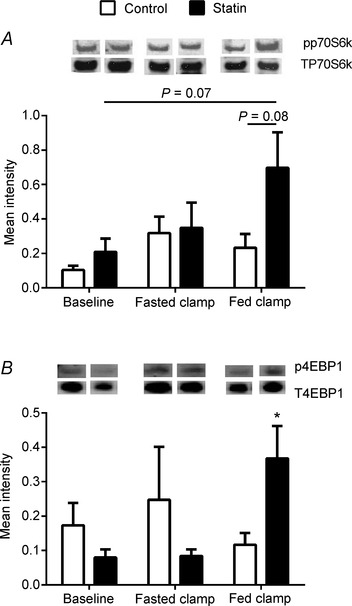
**Expression levels of proteins involved in translation initiation of muscle protein synthesis in skeletal muscle in control and statin user groups** *A*, ratio of muscle phosphorylated to total p70S6k protein expression; and *B*, ratio of muscle phosphorylated to total 4EBP1 protein expression. *P < 0.05 compared to control within the fed state clamp. Values are expressed as mean intensity (normalised to β‐actin) ± SEM.

### Taqman low density array analysis

Following SAM analysis, mRNA expression of 33 genes was significantly (*P *< 0.05) up‐regulated in the muscle of the statin myalgic subjects compared to control when employing a 5% FDR, and no genes were found to be down‐regulated. The major pathways up‐regulated were proteolysis and apoptosis (Fig. 7 *A*), CHO and fat metabolism (Fig. 7 *B*), cell migration and proliferation (Fig. 7 *C*), transcription factors (Fig. 7 *D*) and insulin signalling (Fig. 7 *E*). The magnitude of the fold increase was generally modest (ranging from 1.1‐ to 1.8‐fold greater than control), but six genes were found to be up‐regulated using a 1% FDR. These genes were MMP2, RhoB, BCL2, SREBF2, myostatin (MSTN) and CK. In the case of CK, a profound mean increase in mRNA expression (89‐fold) was evident compared to control, with all statin myalgic subjects displaying a striking increase in muscle CK mRNA expression relative to control, which ranged from ∼55‐ to 130‐fold (Fig. 7 *F*).

**Figure 7:**
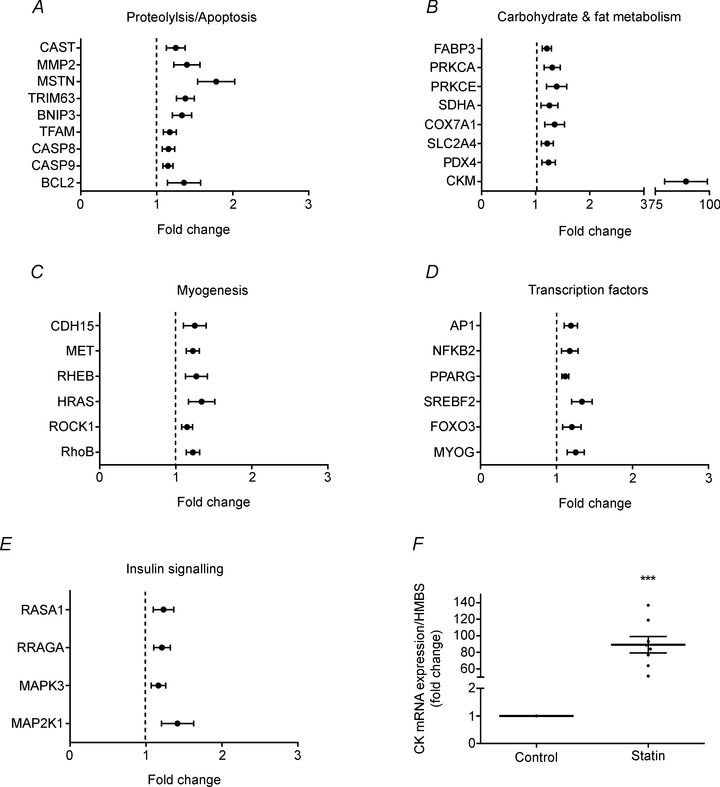
**Muscle mRNA expression normalised to HMBS in the statin user group relative to control at baseline (fold change)** *A*–*E*, SAM analysis, using an FDR of 5%, revealed these genes to be significantly up‐regulated in statin myalgic subjects. Values are expressed as mean ± SEM. Control values are set at 1 and are represented as a dotted line. *F*, muscle creatine kinase mRNA expression normalised to HMBS (fold change relative to control). Values are expressed as mean ± SEM (black horizontal bar) and individual fold difference in statin myalgic subjects (black spots). ***P < 0.001 when compared to control.

## Discussion

To our knowledge, this is the first study to measure muscle protein turnover rates and insulin sensitivity in statin myalgic subjects using gold standard methods. We provide novel and important insight by demonstrating that statin myalgia in older people is not associated with deficits in muscle strength and lean mass or the dysregulation of muscle protein turnover when compared to age‐matched, non‐statin user controls. Furthermore, there were no between‐group differences in systemic or muscle inflammatory markers. However, statin myalgia was associated with a delay in time to peak power output during repeated maximal contractions, increased central adiposity and whole body and leg insulin resistance, and increased expression of muscle mRNAs (which was profound in the case of muscle CK mRNA).

Muscle function in individuals presenting with statin myopathy and myalgia has been examined previously, although the data are equivocal, in that statin myalgic subjects showed either limited changes in muscle function compared to non‐statin users (Parker *et al*. 2012), or exhibited signs of reduced muscle strength leading to an increased risk of falls (Scott *et al*. 2009). The present study revealed no difference in quadriceps isometric strength between control and statin myalgic subjects (Fig. 3 *A*), and accordingly no between‐group difference in leg lean mass (Fig. 2). Similarly, peak power output during 30 maximal knee extensor contractions was not different between groups (Fig. 3 *B*). However, the time taken to reach peak power was greater in statin myalgic than in control volunteers (twelfth contraction *vs*. third contraction, respectively, Fig. 3 *B*), such that the statin myalgic subjects generated significantly less work output per contraction at the onset of exercise (Fig. 3 *C*, *P *< 0.05). This response confirms the presence of exercise intolerance in statin myalgia, most probably due to joint‐ and muscle‐related discomfort at the initiation of exercise. This interpretation is supported by our observation that the magnitude of perceived muscle soreness at rest was 4‐fold greater in the statin user group compared to control (Table 1, *P *< 0.001).

Statin use has been linked to an increased incidence of type 2 diabetes (Preiss *et al*. 2011), and simvastatin in particular has been shown to decrease insulin sensitivity in hypercholesterolaemic patients (Koh *et al*. 2008), as determined by the Quantitative Insulin Sensitivity Check Index (QUICKI), and to impair glucose tolerance during an oral glucose challenge (Larsen *et al*. 2013). The present study is the first to utilise a gold standard assessment of insulin sensitivity (DeFronzo *et al*. 1979) to determine whether statin myalgic subjects present with reduced glucose disposal compared to healthy age‐matched control volunteers. We show that steady‐state whole body glucose disposal under ‘fed state’ insulin clamp conditions was 30% lower in statin myalgic subjects even in the face of greater circulating insulin concentrations (Fig. 4 *A* and *B*). Furthermore, leg glucose uptake was 45% lower in the statin myalgic subjects (Fig. 4 *C*). This lower insulin‐stimulated glucose disposal was not paralleled by increased muscle PDK4 protein expression (Table 4) or blunted activation of muscle PDC (Table 4), clearly suggesting the insulin resistance observed does not reside at the level of PDC activation as has previously been proposed in high fat feeding‐mediated insulin resistance (Mayhew *et al*. 1995). Nevertheless, we did observe increased expression of insulin signalling mRNAs in the statin user group, namely MAPK3, MAP2K1 (Fig. 7 *E*) and FOXO3 (Fig. 7 *D*), suggesting a compensatory adaptation to the heightened insulin resistance observed in these statin myalgic subjects. There is increasing evidence that abdominal adiposity is more closely linked to the development of insulin resistance (Kohrt *et al*. 1993), possibly as a result of increased hepatic fatty acid flux stimulating gluconeogenesis and inhibiting hepatic insulin clearance. Indeed, trunk fat mass was inversely related to whole body glucose disposal rates across all volunteers during the ‘fed state’ insulin clamp in the present study (Fig. 4 *D*). Furthermore, steady‐state serum insulin concentration tended to be greater in the statin myalgic subjects, who also presented with greater abdominal adiposity compared to controls (Fig. 2). These observations cannot, however, explain the lower leg glucose uptake in the statin myalgic group, particularly given leg lean and fat masses were the same.

Reduced physical activity impacts heavily on quality of life, increasing morbidity and overall mortality (Wen *et al*. 2011). There is also increasing evidence that physical inactivity is causative in the development of insulin resistance and type 2 diabetes (Bergouignan *et al*. 2011) and that this can be mediated by a relatively rapid direct effect of inactivity *per se* (Johnson *et al*. 1976), along with a more chronic inactivity‐induced increase in adiposity. The experimental design of the current study does not allow insight into whether the statin myalgic group was more insulin resistant upon entry into the study, whether statin use *per se* was responsible for the insulin resistance observed, or whether a statin‐mediated reduction in physical activity levels because of muscle pain and discomfort was a contributing factor. However, given the increasing incidence of type 2 diabetes with statin use, further research is warranted to determine the relative contribution of each to the aetiology of statin‐associated insulin resistance, particularly given increased physical activity levels are thought to exacerbate statin‐related muscle pain and discomfort (Parker *et al*. 2012). Indeed, emerging evidence from large cohort cross‐sectional and longitudinal studies (Lee *et al*. 2014) does support the view that statin myalgia in older people is associated with lower habitual physical activity, even after accounting for medical history and other potentially confounding factors. Furthermore, there is clear evidence that patients with musculoskeletal dysfunction associated with chronic non‐communicable disease (e.g. chronic obstructive pulmonary disease, osteoarthritis and fibromyalgia) have lower habitual physical activity levels compared to healthy controls (Tudor‐Locke *et al*. 2009). In line with this evidence, the statin myalgic volunteers in the present study presented with increased muscle soreness (Table 1) and a delay in time to peak power output during repeated maximal, voluntary contractions (Fig. 3 *C*). Furthermore, the muscle pain questionnaire used at screening (based on the McGill pain questionnaire; Melzack, 1975) revealed six of the nine statin myalgic volunteers reported walking or lifting as activities that exacerbate their muscular symptoms, and that the alleviation of symptoms occurred with rest and pain medication (Table 2). Collectively, it is not therefore unreasonable to assume that habitual physical activity levels would have been lower than that of the control volunteers in the present study. However, if adiposity and reduced physical activity were indeed principal drivers of the muscle insulin resistance in the statin myalgic group of the present study, one would have expected this cohort to show a blunting of the muscle protein synthetic response to increased nutrient delivery (Breen *et al*. 2013), which was clearly not evident in the present study (Fig. 5 *A*).

Limited studies have examined global gene expression in skeletal muscle of individuals on statin therapy (Urso *et al*. 2005; Laaksonen *et al*. 2006), and none in individuals presenting with overt statin myopathy or myalgia. In the present study, a micro‐fluidic card approach was used to quantify mRNA abundance of genes covering a number of signalling and metabolic pathways in skeletal muscle to shed light on the molecular basis of statin myalgia. Of the 96 mRNAs quantified, 33 were in greater abundance in the statin user group compared to control (Fig. 7; 5% FDR), albeit moderately (1.07‐ to 1.43‐fold increase). None was found to be in less abundance. Twenty‐seven per cent of these 33 differentially expressed genes were found to be involved in proteolysis/apoptosis pathways. This observation concurs with the report of Laaksonen *et al*. (2006) in which high dose simvastatin administration (80 mg day^−1^) resulted in differential expression of 111 genes from predominantly pro‐apoptotic pathways compared to placebo and low dose atorvastatin (40 mg day^−1^) administration, but subjects reported no statin‐related side effects, i.e. muscle pain or serum CK elevation. In the present study, several transcription factors (Fig. 7 *D*) and mRNAs concerned with CHO/fat metabolism (Fig. 7 *B*), myogensis (Fig. 7 *C*) and insulin signalling (Fig. 7 *E*) were also found to be in greater abundance in the statin myalgia group.

Plasma CK and lactate dehydrogenase protein are routinely used as markers of muscle disruption, but the concentration of both was within the normal range in the present study and no difference was observed between experimental groups (Table 1). This was contrasted by the striking between‐group difference in muscle CK mRNA expression, which was between ∼55‐ and 130‐fold greater in the statin user group (Fig. 7 *F*). Increased muscle CK expression is commonly related to mitochondrial myopathies (Stadhouders *et al*. 1994), and in the context of the present study statin administration has been associated with muscle mitochondrial disruption in animal and cell culture experiments (Bouitbir *et al*. 2011), and impaired mitochondrial respiration in human volunteer studies (Larsen *et al*. 2013). Of further note, four of the six genes found to be up‐regulated at the 1% FDR in the present study, RhoB, BCL2, MMP2 and myostatin, have been linked to mitochondrial apoptotic signalling (Bigland & Lippold, 1954). Indeed, it has been suggested that simvastatin (Zörgiebel *et al*. 2007) and myostatin (Bigland & Lippold, 1954) trigger the mitochondrial pathway of apoptosis via translocation of Bax from the cytosol to the mitochondrial membrane, and simvastatin‐mediated up‐regulation of RhoB mRNA and protein levels is paralleled by caspase 2 activation (Zörgiebel *et al*. 2007). In keeping with this, mysotatin knockout mice have abnormal mitochondrial function (Ploquin *et al*. 2012). It may be speculated therefore that the marked upregulation of CK mRNA in the statin myalgic subjects in the present study may have occurred as a protective adaptation against apoptosis, i.e. a compensatory measure to maintain proper cell function (Schlattner *et al*. 2006).

To our knowledge, this is the first study to measure muscle protein turnover rates and associated molecular pathways in statin myalgia. The molecular mechanisms responsible for statin myopathy are poorly defined, particularly *in vivo*. We have previously demonstrated that simvastatin administration inhibits muscle PI3k/Akt signalling, concomitantly with the dephosphorylation (activation) of muscle FOXO transcription factors and increased mRNA abundance of muscle genes thought to regulate proteasomal and lysosomally mediated protein degradation and the suppression of CHO oxidation in a rodent model of statin myopathy (Mallinson *et al*. 2009, 2012). In keeping with this, statin myalgia in the present study was associated with increased expression of genes involved in muscle proteolysis (MuRF1, MMP2), apoptosis (Caspase 8/9, BCL2, RhoB, MMP2, myostatin) and autophagy (TFAM, BNIP3) (Fig. 7). However, these differences in mRNA abundance were modest and did not translate into increased leg protein breakdown, even under post‐absorptive state conditions where the rate of leg protein breakdown would predominate over synthesis (Fig. 5 *B*), or reduced leg lean mass (Fig. 2). It is therefore plausible that the catabolic gene mRNA abundance changes linked to proteolysis, autophagy and apoptosis reflect preliminary stages in the progression from statin myalgia to myopathy (particularly in the case of the latter where four of the six genes up‐regulated at a 1% FDR have been previously linked to mitochondrial apoptotic signalling). Clearly, longitudinal studies are warranted to interrogate this suggestion in greater detail.

In keeping with the post‐absorptive leg protein breakdown responses and lack of difference in muscle mass between statin myalgia and control volunteers, the post‐prandial increase in muscle protein synthesis was not blunted in statin myalgia, and contrasts what might have been expected based on the disruption of PI3k/Akt signalling in rodent statin myopathy (Mallinson *et al*. 2009, 2012). Indeed, statin myalgic subjects in the present study showed no inactivation of the anabolic signalling proteins, p70S6k (Fig. 6 *A*) and 4EBP1 (Fig. 6 *B*), but paradoxically showed increased phosphorylation of 4EBP1 and p70S6k in response to feeding. The greater phosphorylation status of p70S6k and 4EBP1 at the end of the fed state clamp in the statin user group relative to control probably reflects the trend for steady‐state serum insulin concentration to be greater in the statin group (Fig. 4 *A*). We have previously demonstrated this association between the phosphorylation status of p70S6k and 4EBP1 and steady‐state serum insulin concentration, which can be dissociated from the muscle protein synthetic response to a fed state condition (Greenhaff *et al*. 2008). The expression levels of muscle myogenic mRNAs were upregulated in statin myalgic subjects, for example RhoB, HRAS, CDH15 (Fig. 7 *C*) and myogenin (Fig. 7 *D*). However, again the magnitude of the increase, although significant, was small and was not associated with between‐group differences in muscle mass or protein synthetic responses. Of note, however, after CK, myostatin was the most abundantly expressed gene in the statin myalgic subjects, and remained significantly upregulated when using a 1% FDR. Myostatin functions as a negative regulator of satellite cell proliferation and differentiation, and muscle growth and development (Lee & McPherron, 2001). Moreover, increased myostatin mRNA expression appears to be a common feature of human muscle wasting in critical illness (Constantin *et al*. 2011) and chronic muscle disuse (Reardon *et al*. 2001). Whilst this is the first study to demonstrate increased myostatin mRNA expression in statin myalgia, the statin myalgia group showed no evidence of elevated muscle protein breakdown or lower lean body mass. However, myostatin has also been associated with the regulation of muscle insulin sensitivity, with myostatin‐deficient mice exhibiting increased insulin sensitivity (through activating the AMP‐activated protein kinase signalling) (Zhang *et al*. 2011), which is in agreement with insulin resistance being greater in the statin myalgic group of the present study. Indeed, it has recently been demonstrated that increasing myostatin potently induces hepatic and muscle insulin resistance in rodents by degrading IRS1 protein (Bonala *et al*. 2014) and reducing signalling through the Akt/p70S6k pathway in myotubes (Trendelenburg *et al*. 2009).

Finally, it has recently been suggested that glycine amidinotransferase (GATM), a gene that encodes the rate‐limiting enzyme in creatine synthesis, may be the functional link between statin‐mediated lowering of cholesterol and susceptibility to statin‐induced myopathy (Mangravite *et al*. 2013). The authors found GATM to be associated with the incidence of statin‐induced myotoxicity in two separate populations. Moreover, GATM knockdown in hepatocyte‐derived cell lines attenuated the transcriptional response to sterol depletion. This suggests a potential association between creatine synthesis and statin myopathy. However, we found no difference in muscle total creatine or muscle ATP content between control and statin myalgic subjects (Table 1). Furthermore, given creatine synthesis is localised to the liver, it is difficult to rationalise the association between GATM and statin‐related muscle problems, particularly when muscle total creatine stores appear to be normal in statin myalgia (Table 1).

In summary, we have shown the presence of a delay in the time to reach peak power output in older, male chronic statin myalgic subjects compared to age‐matched volunteers, but there was no evidence that this phenomenon was linked to the presence of systemic or muscle inflammation or the disruption of muscle protein turnover and the loss of muscle mass and strength. However, statin use was associated with increased abdominal adiposity, insulin resistance and evidence supporting the advent of mitochondrial dysfunction and apoptosis, which warrants further investigation particularly in relation to statin myalgia potentially reducing habitual physical activity levels and therefore increasing cardiovascular risk.

## Additional information

### Competing interests

No competing interests exist.

### Author contributions

J.M.: collection, analysis and interpretation of data, writing article. K.M.: collection of data, revision of article. A.M.: collection of data, revision of article. A.S.: collection of data, revision of article. K.S.: collection of data, revision of article. D.C.: conception and design of the experiments, revision of article. M.R.: revision of article. P.L.G.: conception and design of the experiments, revising article critically for important intellectual content.

### Funding

This work was supported by The Dunhill Medical Trust (grant number: R101/0209).
